# Machine learning and explainable artificial intelligence to predict and interpret lead toxicity in pregnant women and unborn baby

**DOI:** 10.3389/fdgth.2025.1608949

**Published:** 2025-05-30

**Authors:** Priyanka Chaurasia, Pratheepan Yogarajah, Abbas Ali Mahdi, Sally McClean, Mohammad Kaleem Ahmad, Tabrez Jafar, Sanjay Kumar Singh

**Affiliations:** ^1^ School of Computing, Engineering & Intelligent Systems, Ulster University, Londonderry, United Kingdom; ^2^ Department of Biochemistry, King George Medical University, Lucknow, India; ^3^ School of Computing, Ulster University, Newtownabbey, United Kingdom; ^4^ Department of Biochemistry, Era University, Lucknow, India; ^5^ Department of Computer Science and Engineering, Indian Institute of Technology (BHU), Varanasi, India

**Keywords:** machine learning, classification, predictive modelling, explainable AI, lead toxicity

## Abstract

**Introduction:**

Lead toxicity is a well-recognised environmental health issue, with prenatal exposure posing significant risks to infants. One major pathway of exposure to infants is maternal lead transfer during pregnancy. Therefore, accurately characterising maternal lead levels is critical for enabling targeted and personalised healthcare interventions. Current detection methods for lead poisoning are based on laboratory blood tests, which are not feasible for the screening of a wide population due to cost, accessibility, and logistical constraints. To address this limitation, our previous research proposed a novel machine learning (ML)-based model that predicts lead exposure levels in pregnant women using sociodemographic data alone. However, for such predictive models to gain broader acceptance, especially in clinical and public health settings, transparency and interpretability are essential.

**Methods:**

Understanding the reasoning behind the predictions of the model is crucial to building trust and facilitating informed decision-making. In this study, we present the first application of an explainable artificial intelligence (XAI) framework to interpret predictions made by our ML-based lead exposure model.

**Results:**

Using a dataset of 200 blood samples and 12 sociodemographic features, a Random Forest classifier was trained, achieving an accuracy of 84.52%.

**Discussion:**

We applied two widely used XAI methods, SHAP (SHapley additive explanations) and LIME (Local Interpretable Model-Agnostic Explanations), to provide insight into how each input feature contributed to the model’s predictions.

## 1 Introduction

Lead, a global pollutant, has been tracked in every aspect of environmental and biological systems (1). Lead is a neurotoxin that influences human health, including birth outcomes and child development. The global death rate linked to high Blood Lead Level (BLL) has increased steadily by 21% (2). In 2019, lead poisoning caused more than 900,000 premature deaths globally (1.6% of overall deaths), which is comparable to the number of deaths caused by HIV/AIDs (3).

Pregnancy is a critical time for lead exposure to the mother and foetus (4). Lead crosses the placenta freely (5) causing complications in foetal growth. Lead can negatively impact a range of birth outcomes, by accumulating in the placenta and causing oxidative stress, reduced nutrient transfer, and abnormal function (6). Poor birth outcomes are known to be linked with poor developmental trajectories in infants, leading to long-term impact on adult health (4). Lead toxicity causes increased risk of kidney, cardiovascular and liver disease later in life (7). Lead affects the developing foetus and the pregnant woman (8). During pregnancy, lead in maternal blood can cause miscarriage, spontaneous abortions, gestational hypertension (9), congenital malformations, and stillbirths (8). One of the major silent killers due to lead poisoning is pre-eclampsia, which is significantly related to maternal ill health and mortality (10).

Children are particularly exposed to the neurotoxic effects of lead and even small levels of lead exposure can cause serious and, in a few cases, permanent neurological damage. Children are not only born with lead transferred to them from their mothers but also have greater lead exposure as their growing bodies absorb more lead than adults do (4). UNICEF reported that 1 in 3 children (i.e., nearly 800 million) globally have BLL at or above 5 μg/dL, a threshold that the Centres for Disease Control and Prevention has commended to initiate action (11). Nevertheless, there are no safe lead levels and the harmful effects of lead appear at all levels. As a result, the economic costs associated with childhood lead exposure are substantial (12). The World Bank analysis reported that in 2020, the annual costs of childhood lead exposure are estimated to be at least $50.9 billion in the US, $55 billion in the EU, and $977 billion in low-middle-income countries (11). On the contrary, the economic gain accomplished by effective interventions against lead poisoning is substantial (13). The benefits gained by effective lead toxicity management far outweigh the costs of creating a national lead screening, prevention, and surveillance programme. Both UNICEF and Pure Earth have insisted countries strengthen their healthcare systems to tackle the effects of lead poisoning (1). An upgrade in the existing measures should involve more active lead screening, monitoring, and reporting approaches. In the current approach, a lab-based blood test is required to determine lead poisoning. The limitation of this approach is that it requires an expert medical/technical staff, blood samples and expensive resources like atomic absorption spectrometry. As a result, this approach is inappropriate for doing early screening in a larger population.

Keeping the above views in mind, it is necessary to identify the features that contribute to maternal BLL, particularly those that could be minimised to reduce the transfer to the developing foetus. Previous research in this area has identified multiple features that contribute to maternal lead exposure. It was found that lead levels are higher in pregnant women who are more exposed to environmental contamination. The lead levels are higher in mothers exposed to environmental contamination, particularly areas of high pollution and those living near lead-based industries (3). In addition to environmental factors, there are added sociodemographic features that directly or indirectly contribute to elevated lead levels. Sociodemographic features such as water, dust, soil, occupational and take-home exposure highly contribute to lead poisoning (14–16). The use of cosmetics additionally contributes to lead poisoning in a pregnant woman (17). Even though research has established a relation between lead poisoning in women and sociodemographic features (4, 18), the magnitude to which these features affect the level of lead poisoning remains unexplained. Hence, quantifying sociodemographic factors and explaining the effects can help take timely abatement measures in lead-exposed women. Henceforth, minimise the harmful impact of lead on the developing foetus.

In this context, the “safe motherhood intervention” project is proposed to deliver a low-cost point-of-care analytical tool. The tool will be a computational model in the form of a mobile-based application (app) that could predict lead levels in maternal blood without the need for lab testing as a first instance of finding lead exposure. The work is directed towards increasing the understanding of the factors that contribute to lead levels in pregnant women and the objectives of the project are: (1) Identify a set of sociodemographic features that are lead exposure pathways for a pregnant woman, (2) Develop an easy-to-access and non-invasive set of questionnaires based on the identified features, (3) Collect maternal data consisting of blood samples and questionnaires, (4) Perform data analysis on the collected data and find the optimal set of features that support or do not support lead prediction modelling, (5) Estimate the underlying function and build the computational model, while keeping the size of the resulting model small and easy to interpret, (6) Evaluate the built model performance in predicting the lead toxicity level based on the set of input features, and (7) Design and develop the mobile-based screening app with the embedded prediction model.

The project is novel in creating a truly interdisciplinary project and utilising the benefits of mobile technology and ML techniques. The project aims to reach out to a larger population with the aid of technology. In our previous work, through the collaboration between Ulster University, UK, and the Indian universities: King George Medical University and Era University, maternal data is collected. Following, data analysis and feature selection, a 12-feature set was selected as an optimum set of features for building the lead prediction model (19–21). Further details of the obtained results from this work are described later in this paper.

In the initial work, we demonstrated the possibility of using ML techniques for predicting lead levels in pregnant women using a set of sociodemographic features. From our initial work, we know that the 12-features-based lead prediction model gave the best performance accuracy; however, we don’t know to what extent each feature individually contributed to building the prediction model. In this context, there is a general distrust among stakeholders in using the ML models. These models are considered black boxes whose results are difficult to understand and interpret. The built lead prediction model is expected to demonstrate transparency and explain the rationale behind the predictions. Therefore, it is desired that the built models can explain the prediction made and correctly quantify the level to which its decisions are reliable (22). The growing complexity of ML models has led to growing scientific interest in XAI. In this paper, we demonstrate a first attempt to use XAI to explain the outcomes predicted by the lead prediction model. In this paper, a web-based application is also developed as an interface to display the outcome of ML prediction and XAI results. The interface helps in comprehending all the results at one point for interpretation.

## 2 Methodology

This section describes the previous work and XAI in brief.

### 2.1 Initial work

**Figure 1:**
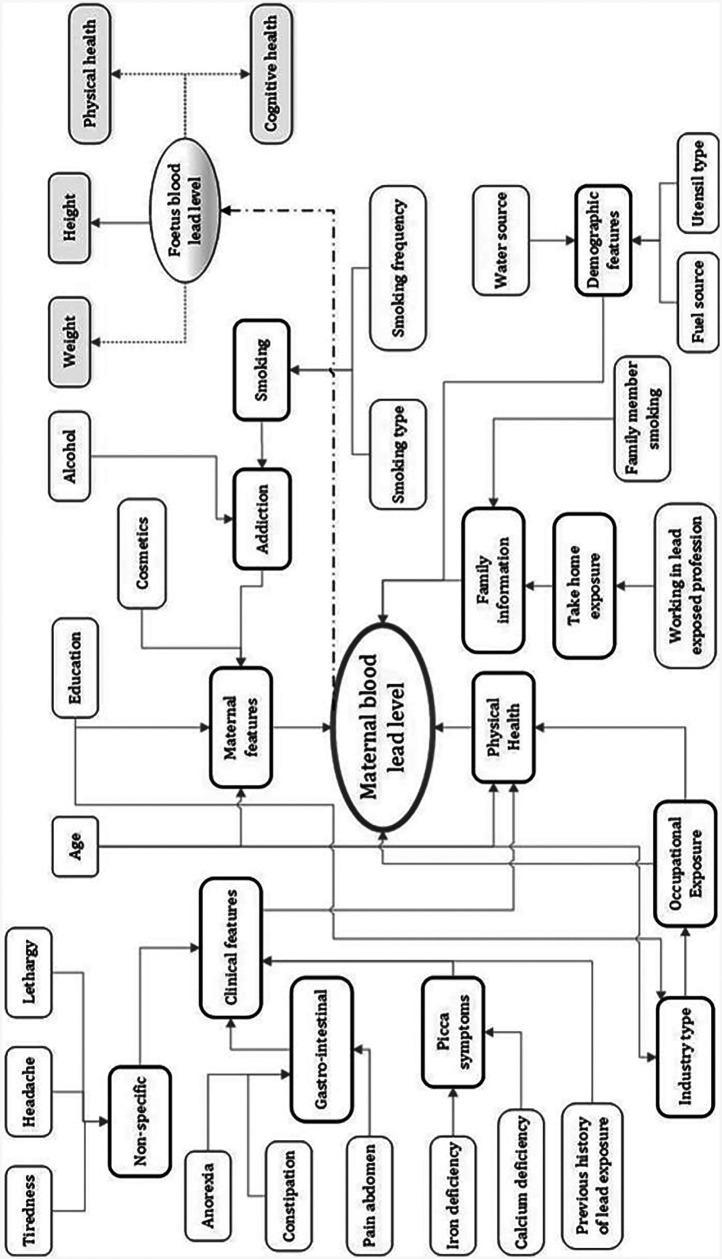
Influence diagram showing features that impact maternal blood lead levels. Reproduced with permission from “Influence diagram of features impacting maternal BLL and toxicity exposure” by Priyanka Chaurasia, Sally I. McClean, Abbas Ali Mahdi, Pratheepan Yogarajah, Jamal Akhtar Ansari, Shipra Kunwar and Mohammad Kaleem Ahmad, licensed under CC BY 4.0.

At the start of the project, sociodemographic features related to maternal lead toxicity levels were identified through interviews with the research and development team which included neonatologists, gynaecologists, research nurses, biomedical engineers and computer scientists. The sociodemographic features included information linked to the maternal’s lifestyle and environmental factors. An influence diagram detailing the features that may have a direct/indirect influence on maternal lead toxicity levels and also the foetus’s lead toxicity level is designed (19–21). Figure 1 shows the influence diagram detailing the set of features that affect maternal lead exposure and consequently affect the child. The influence diagram explains the interconnections between the features themselves and more notably, the link with the maternal’s BLL. The diagram has independent features (enclosed within the rectangles with thin lines) and summary features (enclosed within the rectangles with thick lines). It is to be noted that the independent features can influence summary features and summary features could be independent features in their own right. For example, in Figure 1, the independent features, age and education are likely to impact the subject’s industry type (a summary feature), which consecutively can impact the subject’s occupational exposure (a summary feature) to lead. Figure 1 additionally shows the apparent effect of maternal lead toxicity on the developing foetus. The lead in maternal blood impacts the baby’s weight, height, and physical and cognitive health.

Based on the identified set of features, a questionnaire form was designed. The maternal data comprising blood samples and 18 sociodemographic features were collected from 200 pregnant women at Era’s Lucknow Medical College and Hospital, India (23). The blood samples were analysed and BLL per sample was calculated. In the collected data, the BLL values ranged between 2.3 μg/dL to 34.8 μg/dL. The sample also contained not detected (ND) values, which were recorded for those cases where lead was not detected in the blood sample. The measured BLL samples formed the output class, *lead content level*, and were categorised into four labels: ND_5 (BLL values not detected or less than 5 μg/dL), Between5_10 (BLL values between 5 μg/dL and 10 μg/dL), Between10_15 (BLL values between 10 μg/dL and 15 μg/dL) and GreaterThan15 (BLL values greater than 15 μg/dL). The data was discretised to avoid variability in the data (24, 25) resulting in each feature having a nominal value. The built computational model can take in a set of input features such as personal, take-home exposure and clinical features and then can predict the output class variable, *lead content level*. With this aim, the collected data were pre-processed, and data imbalance was handled. Following feature selection methods, a 12-feature set (Table 1) obtained from the Boruta algorithm gave better prediction results (*k* Nearest Neighbour (*k*NN) = 76.84%, Decision Tree (DT) = 74.70%, and Neural Network (NN) = 73.99%) (21). All the models were built in WEKA Experimenter (University of Waikato, Version 3.8).

**Table 1:** Significant features obtained by applying the Boruta algorithm to the four-label dataset (21).

Feature	Details
Age	Age of the mother: lessThanEqual30, gretaerThan30
Lipstick	Use of lipstick: Yes, No
Kohl	Use of kohl: Yes, No
Sindoor	Use of sindoor: Yes, No
Education	Highest education level
Water source	Source of water: ground, reverse osmosis (RO), tap water
Utensils	Type of utensils used: steel, aluminium, ceramic
Take-home exposure	Lead exposure due to family member/s working in lead-based industries
Occupational exposure	Housewife, office
Pica symptoms	Calcium deficiency, iron deficiency
Gastrointestinal	Anorexia, constipation, pain abdomen
Non-specific	Headache, lethargy, tiredness

### 2.2 Why XAI in lead toxicity prediction?

AI is more pervasive now and the need for interpretability and transparency in AI-based systems is increasingly growing (22). Most of the AI systems are complex and act as a black box (26–28), leading to lower trust among clinicians and end-users. Additionally, these systems offer limited system support in summarising the diagnoses, as they don’t give any explanation of the reason why a particular prediction was made (28). With black-box AI models lacking transparency, it is essential that these models unbox the decision made (22) and make AI systems more explainable, transparent, and auditable.

**Figure 2:**
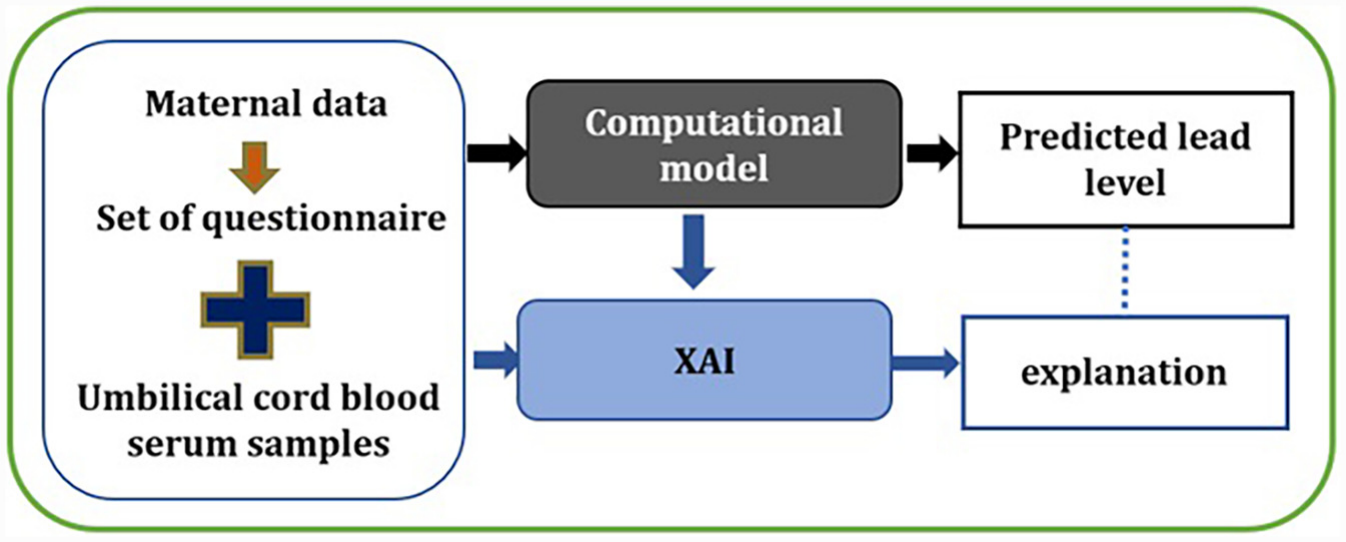
XAI framework for automated lead toxicity prediction model.

With the use of XAI techniques, it is possible to understand model predictions. XAI with a set of frameworks explains how the AI model made a particular prediction (29). This can help in developing trust and reliability in the healthcare systems, accelerating disease diagnosis, and meeting adherence to regulatory requirements (22). Furthermore, improving trust, transparency, explainability and fairness can also help in enhancing the built model performance by supporting an understanding of its possible weaknesses. Knowing how and why the model works and why it fails sometimes can help in improving and optimising the models as well. The consequences of both false positive and negative cases have an impact on individual welfare and cannot be ignored. For example, in our case, if the lead prediction model incorrectly suggests that a pregnant woman has a high level of lead toxicity. This prediction can cause a negative impact on the subject along with the associated financial cost in further diagnoses and treatment. On the other hand, if a potential subject having high lead toxicity is missed by the model, the whole idea of intervention stands invalid as a potential chance is missed for early intervention in this case. Additionally, when we aim to take pre-emptive measures and adjust modifiable risk factors, we should know which factors are contributing to a high level of lead prediction in a particular subject. For example, from our previous work described in the previous section, we know that features detailed in Table 1 are significant factors in accurately classifying the lead toxicity level. However, not all the features will equally contribute to the model making a particular prediction. Therefore, incorporating an XAI framework as an interpretability layer on top of the lead prediction model can assist in understanding and trusting the decision made by the model and which features contributed more significantly to the predicted lead toxicity levels. Based on the explainability of features, a focus could be made on minimising the modifiable risk factors. Figure 2 details the methodological overview of this work. The XAI layer is added to the lead prediction model that is built from the maternal data. The model predicts the lead level and the XAI framework explains the predictions made. For this work, we used two of the popular XAI frameworks, SHapley Additive exPlanations (SHAP) [30] and Local Interpretable Model-Agnostic Explanations (LIME) [31].

SHAP is an XAI method to explain individual predictions. SHAP explains the individual prediction by computing the contribution of each feature in making the prediction. It computes SHAP values, which are used to determine the impact of each feature on the prediction made (30). SHAP values provide details for each instance, and how a particular feature distributes the prediction results (output) among the features. A SHAP value helps to quantify a feature’s contribution towards the prediction made (30). For a given instance, the built ML model predicts the output and the SHAP explains that the features with a positive SHAP value contributed more towards a particular prediction and the negative values do the opposite. SHAP values (*n*, *m*), denote the average contribution of each feature to the prediction made by the model, where *n* is the number of samples and *m* is the feature matrix (30). LIME is an XAI method to provide local model interpretability. The local aspect in LIME means that it is used to explain individual predictions of a ML model. LIME amends a single data instance by tweaking the feature values and examines the resulting impact on the output (31).

## 3 Results

This section describes the built models and the explanation of lead toxicity prediction.

### 3.1 Lead prediction modelling

For using the XAI framework, we rebuilt the models in Python using the 12-feature dataset and four class labels. In the collected dataset of 200 samples, the output class variable, *lead content level*, has a varying number of instances for each of the class categories: ND_5 (105), Between5_10 (52), Between10_15 (14), and GreaterThan15 (29). The collected dataset is imbalanced and this imbalance in the class sizes can affect the outcome of some of the classification algorithms, usually with a bias towards the majority class (i.e., the class which has a higher number of instances in the dataset) (32). To avoid this bias and handle imbalance, the dataset is resampled. The aim of rebalancing the data is to let the model classify the new observation solely based on the robustness of the algorithm and the merits of the features. The Synthetic Minority Over-Sampling Technique (SMOTE) is applied to the data. The application of SMOTE led to 420 samples in total, with nearly 105 instances of each class. An 80/20 sample split was done, dividing a dataset into two subsets, with 80% of the data (336 instances) used for training and 20% of the data (84 instances) used for testing the model. In our previous work, a range of popular classification algorithms were evaluated for their suitability in the lead prediction task. Six different classification algorithms NN, *k*NN, DT, Adaptive Boosting, Support Vector Machine, and Classification and Regression Trees were applied, out of which NN, *k*NN, and DT gave better prediction results. Therefore, in this work, these three classification algorithms were used. In addition to this, we also applied a Random Forest (RF) classification algorithm to the dataset. Table 2 details the performance of the classification algorithms applied to the rebalanced dataset. From the results, it is observed that the RF-based algorithm gave the best prediction result (84.52%) on the test data. The hyperparameters used for the RF-based model are max_features = 4 and n_estimators = 200.

**Table 2:** Average prediction accuracies, precision, recall and F1-score obtained for different classification algorithms.

Algorithm	Results			
	Class label	Precision	Recall	F1-score
NN	ND_5	0.70	0.76	0.73
	Between5_10	0.83	0.48	0.61
	Between10_15	0.95	0.95	0.95
	GreaterThan15	0.68	0.90	0.78
Average prediction accuracy = 77.38%				
kNN	ND_5	0.65	0.71	0.68
	Between5_10	0.85	0.52	0.65
	Between10_15	0.87	0.95	0.91
	GreaterThan15	0.72	0.86	0.78
Average prediction accuracy = 76.19%				
DT	ND_5	0.80	0.57	0.67
	Between5_10	0.67	0.67	0.67
	Between10_15	1.00	0.95	0.98
	GreaterThan15	0.71	0.95	0.82
Average prediction accuracy = 78.57%				
RF	ND_5	0.77	0.81	0.79
	Between5_10	0.93	0.67	0.78
	Between10_15	1.00	0.95	0.98
	GreaterThan15	0.74	0.95	0.83
Average prediction accuracy = 84.52%				

### 3.2 Explaining lead prediction model

The best model obtained from the lead prediction modelling is the RF-based model with a 12-feature set, with an accuracy of 84.52%. Therefore, we demonstrate the explainability of the RF-based model in predicting the outcome of the test data using SHAP and LIME frameworks.

#### 3.2.1 Summary of feature importance

**Figure 3:**
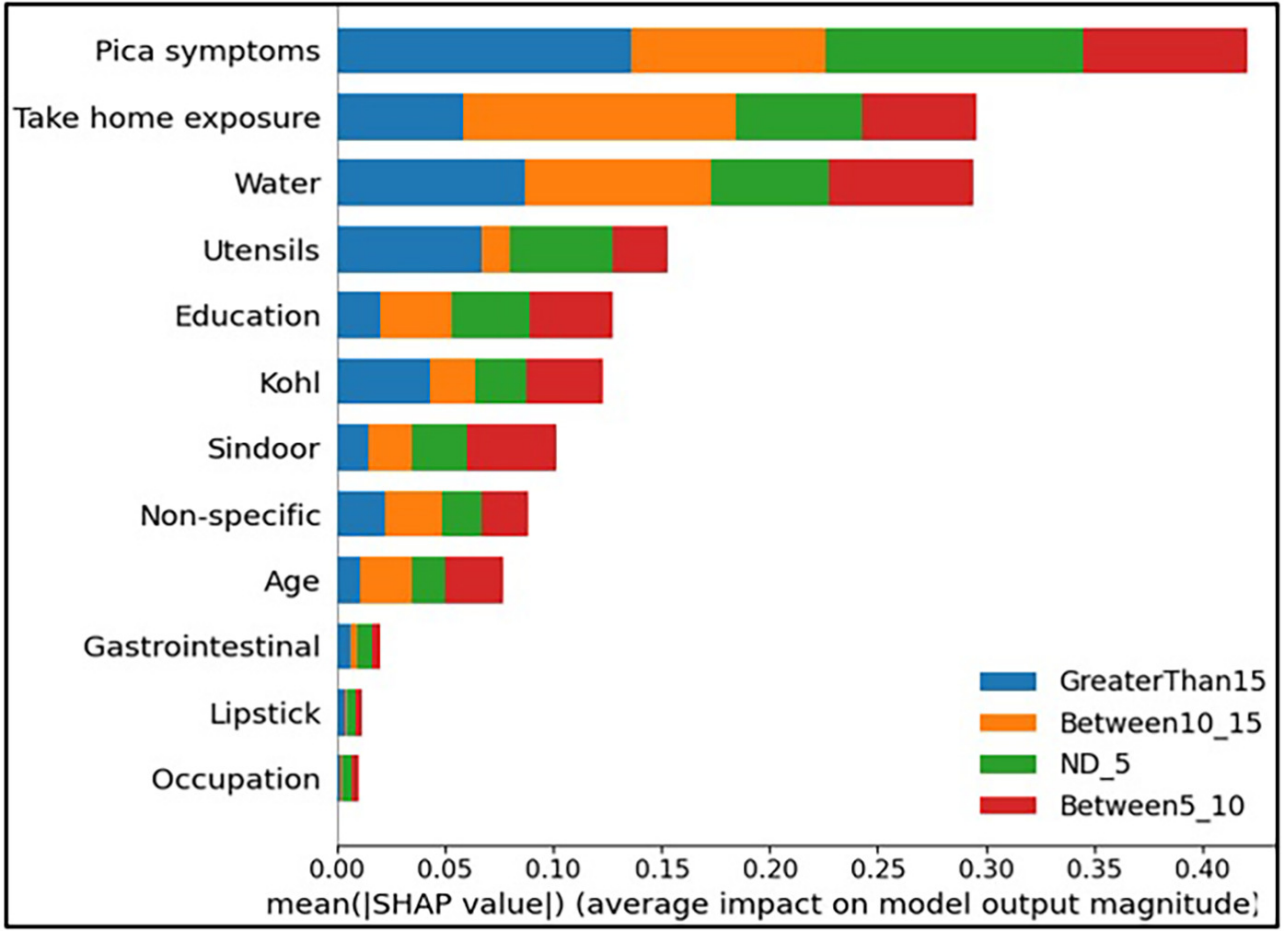
Summary bar plot showing important variables, indicating the magnitude of each feature in the prediction of the class labels in all the instances of the test data.

First, a set of global plots is shown to visualise the overall contribution of a feature over the entire data and how these features influence the output of the built lead prediction model. The global effect of the 12 features is shown in Figure 3 through a summary bar plot. In this plot, the 12 features are evaluated by their average absolute SHAP value; hence if the feature has a positive or a negative influence on prediction does not matter. The features are ranked from the highest to lowest impact on the prediction. The summary bar plot indicates the average impact (magnitude) of each feature in the prediction of the class labels. From the summary bar plot in Figure 3, it is found that the pica symptoms, water, and take-home exposure are the top three features that have the most predictive power.

Existing literature indicates that pica symptoms significantly contribute to lead toxicity. Iron deficiency frequently coexists with lead intoxication and anaemia is a usual symptom of lead poisoning (33). This is observed in our case as well, iron and calcium deficiency resulted in higher levels of lead (GreaterThan15, in our case) as can be seen from Figure 3. The second important feature in determining the class labels is the water source in Figure 3. Water is the source of baseline exposure to lead poisoning (15, 34, 35), and signifies lead exposure pathways to the population at large. The third important feature Take-home exposure. It is found in the existing literature that women are exposed to lead by handling or washing their family members’ lead-contaminated clothes (36). Few of the jobs have potentially high exposure to lead. In our previous work (21), the data analysis indicated that pregnant women whose family members worked in auto repair, auto driving, construction, painting, plastic manufacturing, polishing, pipe fitting, soldering, battery manufacturing and repairing had higher lead concentrations due to take-home exposure. It was found that certain combinations of jobs reflected very high lead take-home exposure in pregnant women. This included Polishing_Soldering (mean BLL = 19.7 μg/dL), Painting_Furniture (mean BLL = 11.05 μg/dL), and Construction_Painting_Plastic_Polishing (mean BLL = 12.6 μg/dL).

**Figure 4:**
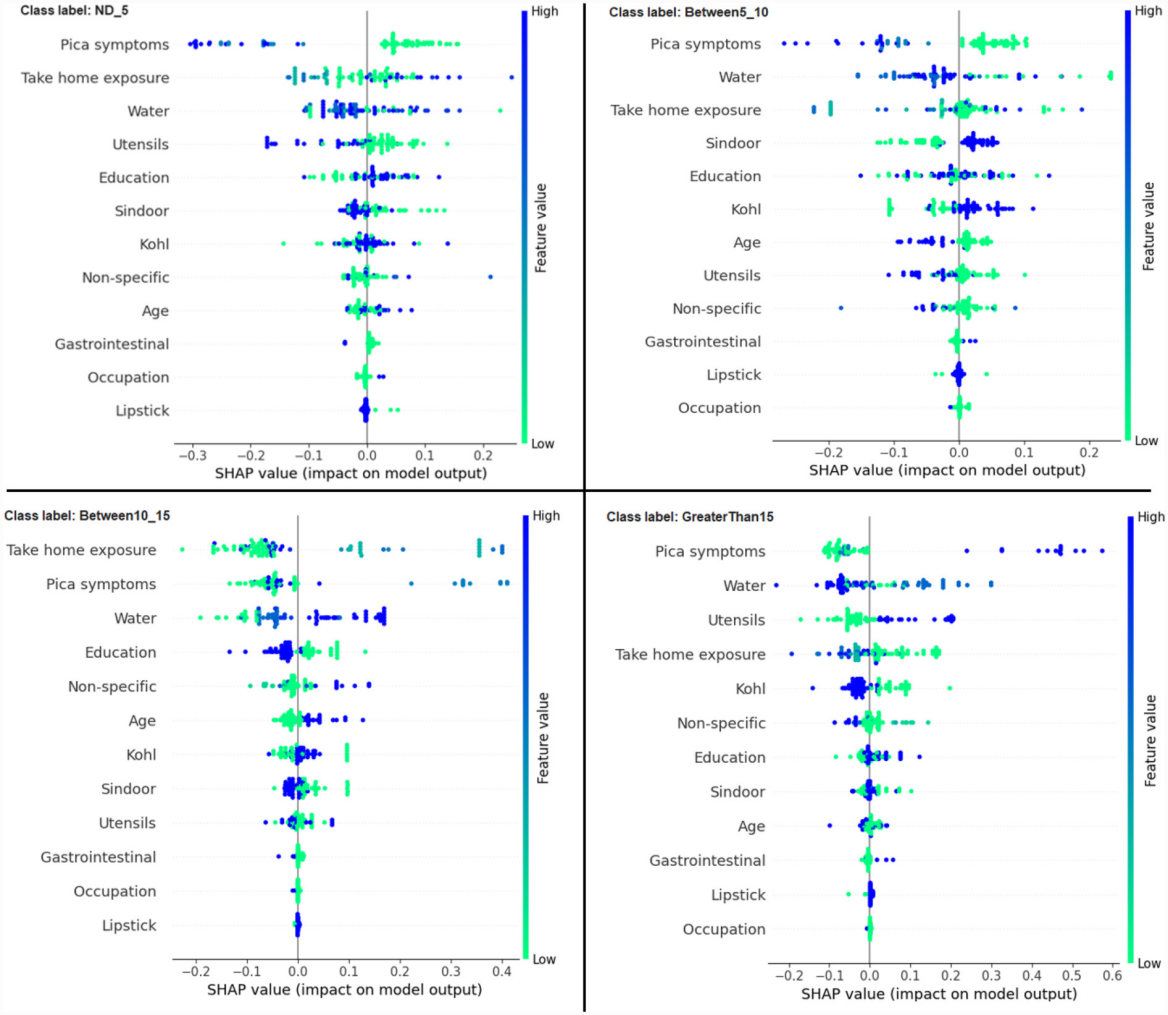
Beeswarm summary plots of representative SHAP values for the 12 features in our model, from most significant to the least significant one (top to bottom) for each of the class: ND_5, Between5_10, Between10_15 and GreaterThan15.

Another global plot, the beeswarm summary plot is shown in Figure 4 for each class label ND_5, Between5_10, Between10_15, and GreaterThan15. The little dots on the plot in Figure 4 correspond to an individual data instance (a single observation). The horizontal axis represents the SHAP value, and the colour of the dots indicates if that observation has a lower (green dots) or higher (blue dots) SHAP value than other observations. The *x*-axis shows the positive or negative influence of the feature on the predicted class label. The SHAP values toward the left have a “negative” effect and SHAP values toward the right have a “positive” effect on the output class label. In the top left figure in Figure 4, it is observed that the lower SHAP value for pica symptoms (green dots) results in the model classifying the observation as class label ND_5. In the bottom right figure, a higher SHAP value of pica symptoms results in the model classifying the observation as class label GreaterThan15. This indicates that the subjects having no pica symptoms have low lead exposure (ND_5), whereas those having pica symptoms have a high lead toxicity level (GreaterThan15). Hence, by using the SAHP global plot, we have the visibility of what are the top significant feature out of 12 features.

#### 3.2.2 Individual interpretation

Next, we show how each of the 12 features contributed to predicting a particular output class for a given observation. Here we show four typical examples to illustrate the interpretability of the RF-based model for each of the output class labels: ND_5, Between5_10, Between10_15, and GreaterThan15, using SHAP and LIME frameworks.

**Figure 5:**
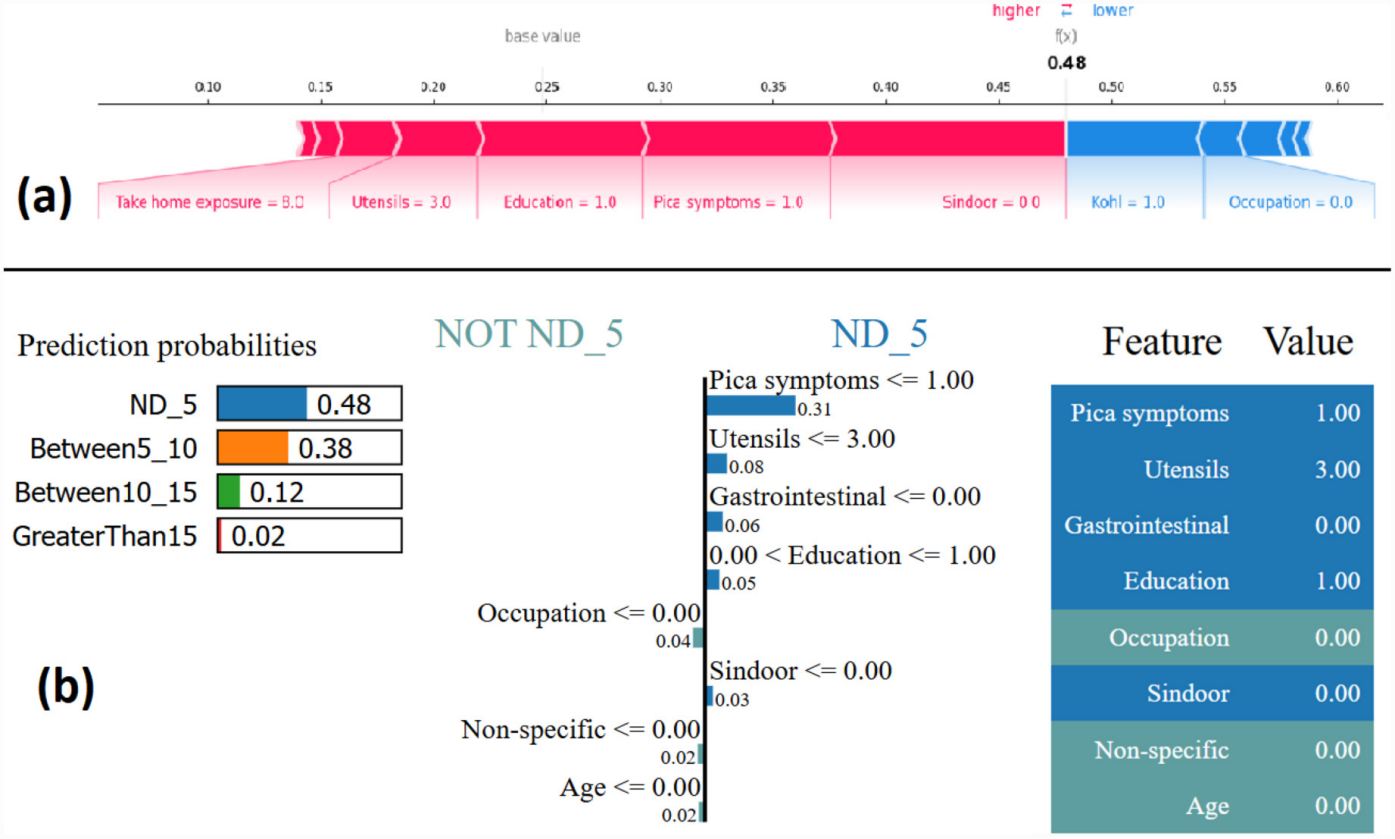
Example of the output class ND_5: (**a**) Interpretation of model prediction results based on SHAP, (**b**) Explanation provided by the LIME model.

The force plot offers a good summary of the prediction made. The base SHAP values for the class labels are ND_5 = 0.2506, Between5_10 = 0.2469, Between10_15 = 0.2486, and GreaterThan15 = 0.2539, which are the baseline for each of these class predictions. Figure 5a shows the force plot for the given data instance and the predicted class is “ND_5.” The features that are important in making the prediction for the given observation are shown in blue and red in Figure 5a. The blue represents those features that pushed the model to score lower and the red represents those features that pushed the model to score higher. Features that have significantly contributed to making the prediction are situated closer to the dividing boundary between blue and red, and the size of the contribution is denoted by the size of the bar. For the given observation, the prediction score is adjusted based on the SHAP value of each feature. In Figure 5a, the force plot starts with 0.2506 as a base value and then the joint effects of all the features push the value in a positive direction, giving the final value of 0.48. The final value of 0.48 corresponds to the prediction score made for the given observation and the prediction corresponds to the class ND_5.

The output by applying LIME is a list of explanations, showing the contribution of each feature of the given observation. Figure 5b shows the model interpretability for the given observation using LIME. The values of each feature for the given observation are summarised in Figure 5b as a table (far right). The middle figure in Figure 5b shows the list of features that positively and negatively contribute to the prediction of class ND_5. The features on the right side of the vertical line positively contributes to predicting ND_5, whereas the feature on the left side of the vertical line negatively contributes to predicting Not ND_5. The figure on the left side of Figure 5b shows the prediction probabilities for each class. The output class label is assigned as ND_5 based on the highest probability value.

**Figure 6:**
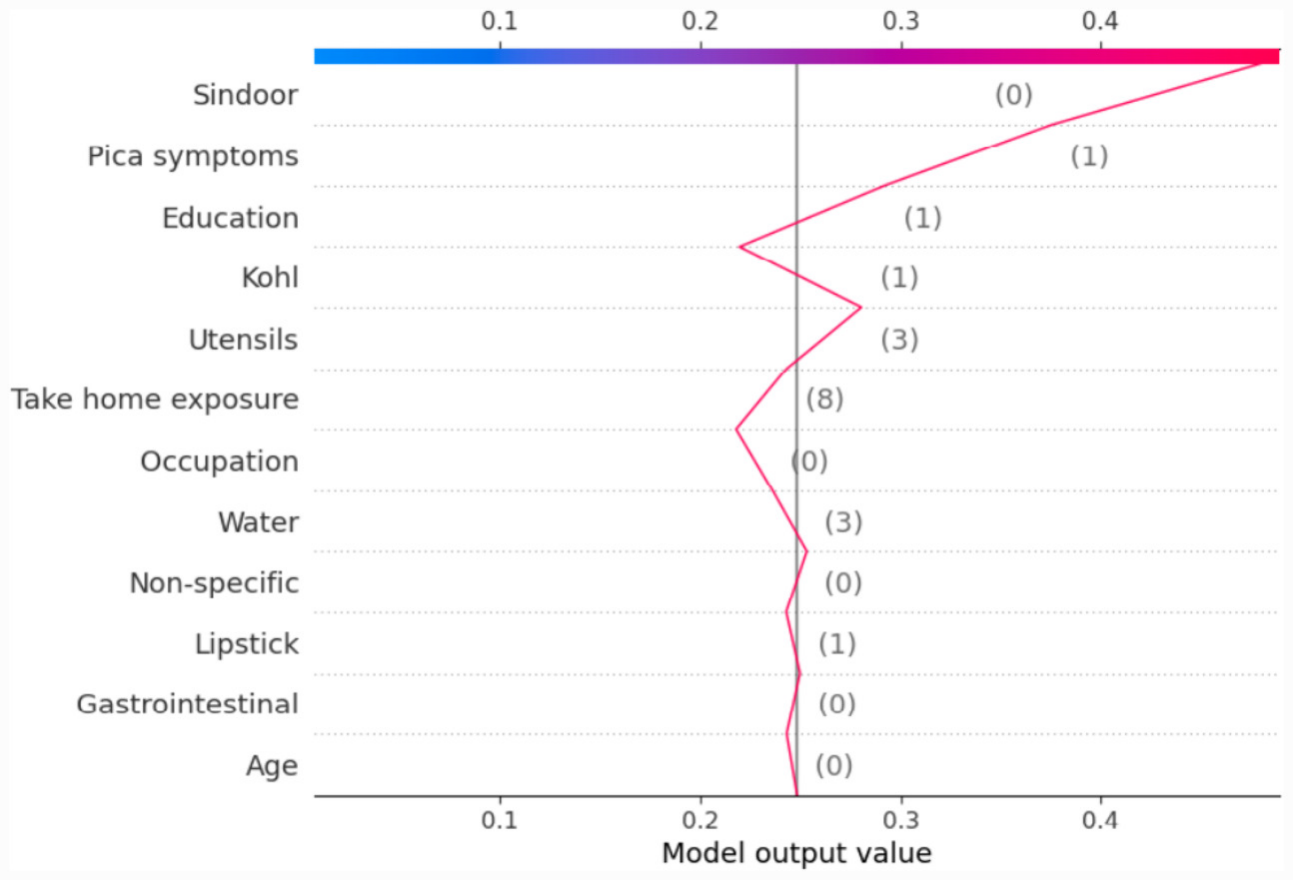
Example of SHAP decision plot for the output class ND_5.

Most of the studies using XAI commonly use only force plot for showing the local interpretability. In this paper, we additionally use the decision plot for understanding and interpreting the predictions made by the model. Both the force plot and the decision plot are effective in explaining the model’s prediction. Nevertheless, the decision plot is more efficient than the force plot. A large number of features can be shown on decision plot. This is useful in those case when a large number of features significantly contribute to the final prediction score. Figure 6 shows the decision plot created for the same given observation shown in Figure 5, for which the predicted output class is ND_5. In the plot, the straight vertical line shows the base value (0.2506) and the coloured line deviating from the straight vertical line is the final prediction. Starting from the base, the prediction line indicates how the SHAP values of each feature add on to reach the final prediction score at the top of the plot.

**Figure 7:**
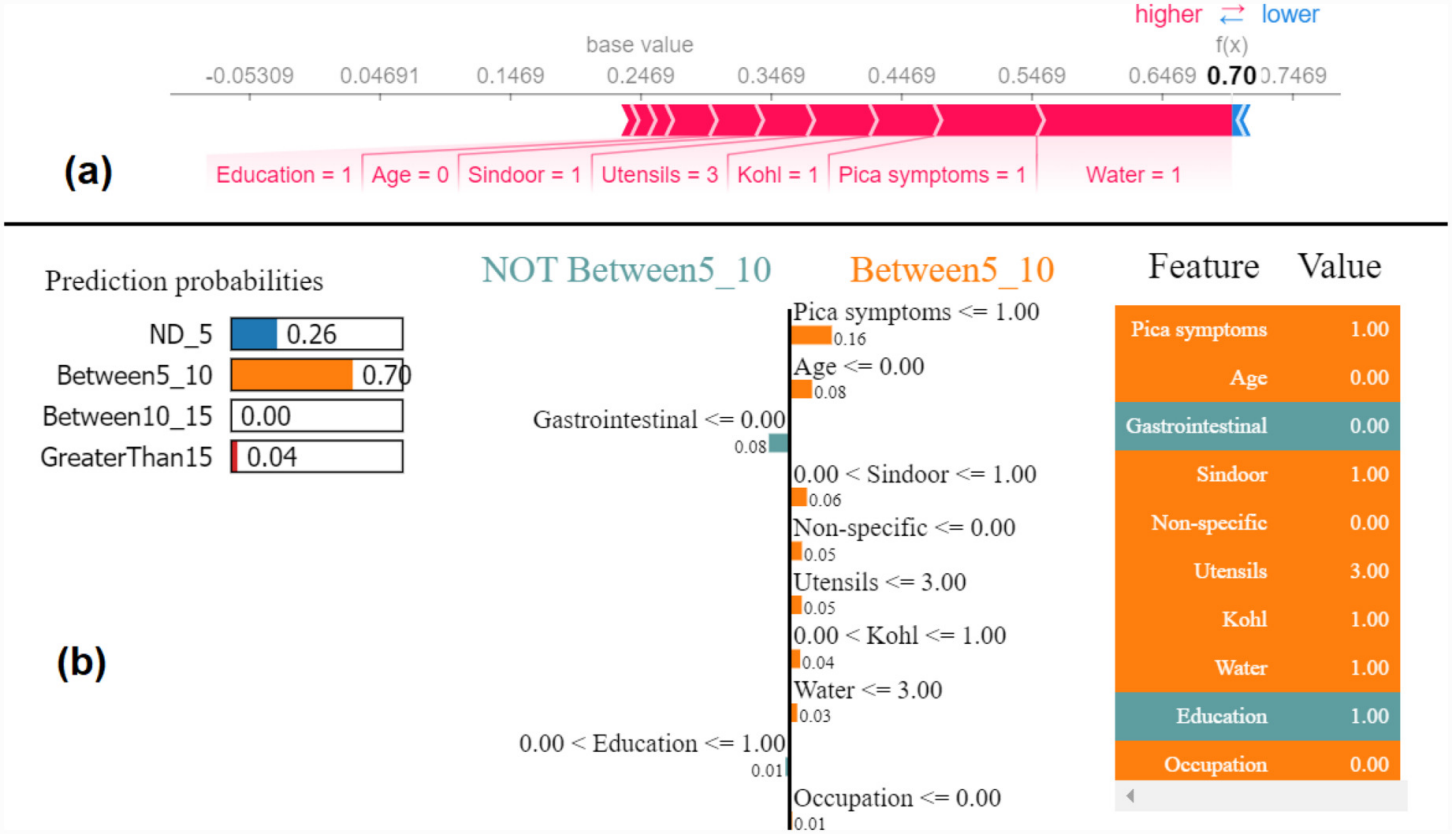
Example of the output class Between5_10: (**a**) Interpretation of model prediction results based on SHAP, (**b**) Explanation provided by the LIME model.

**Figure 8:**
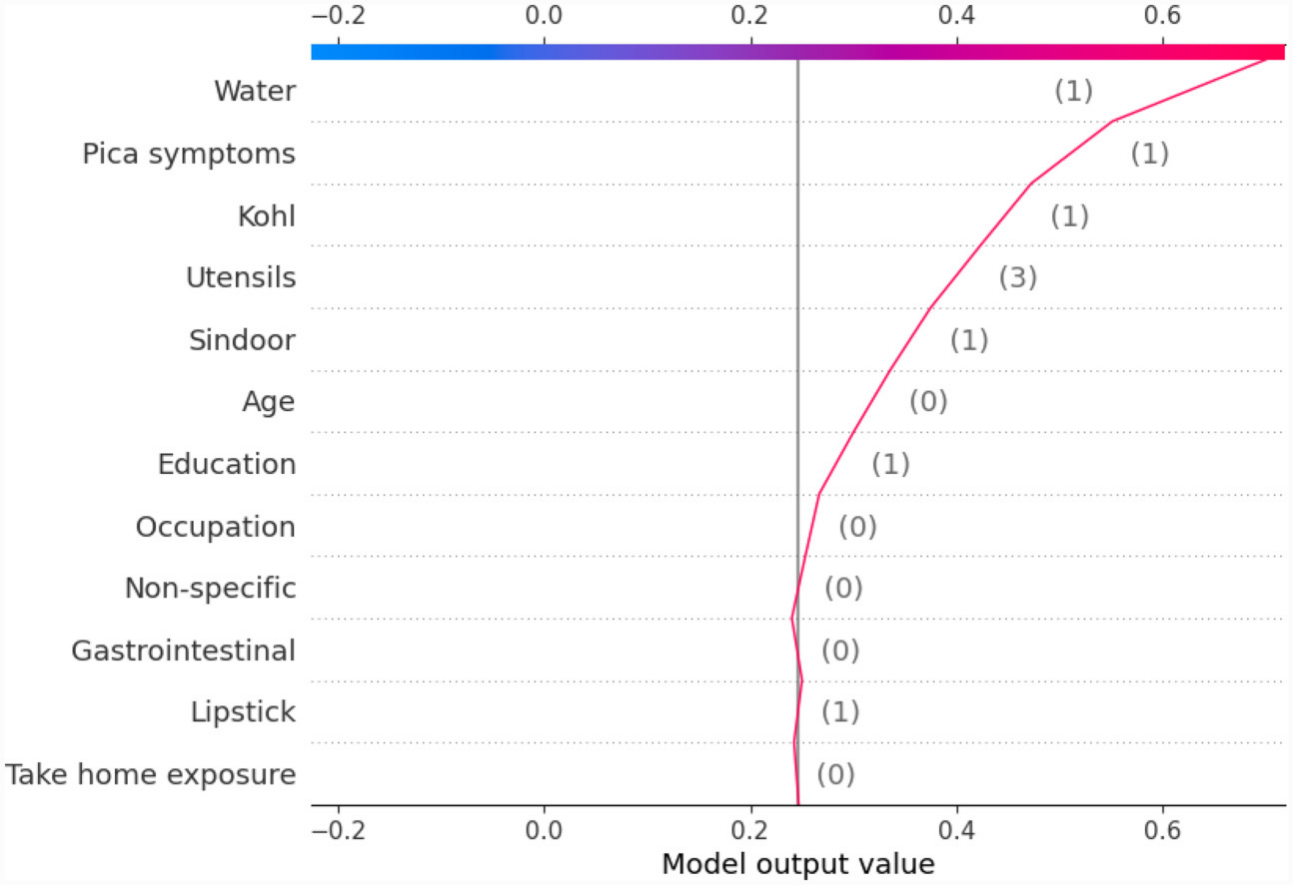
Example of SHAP decision plot for the output class Between5_10.

**Figure 9:**
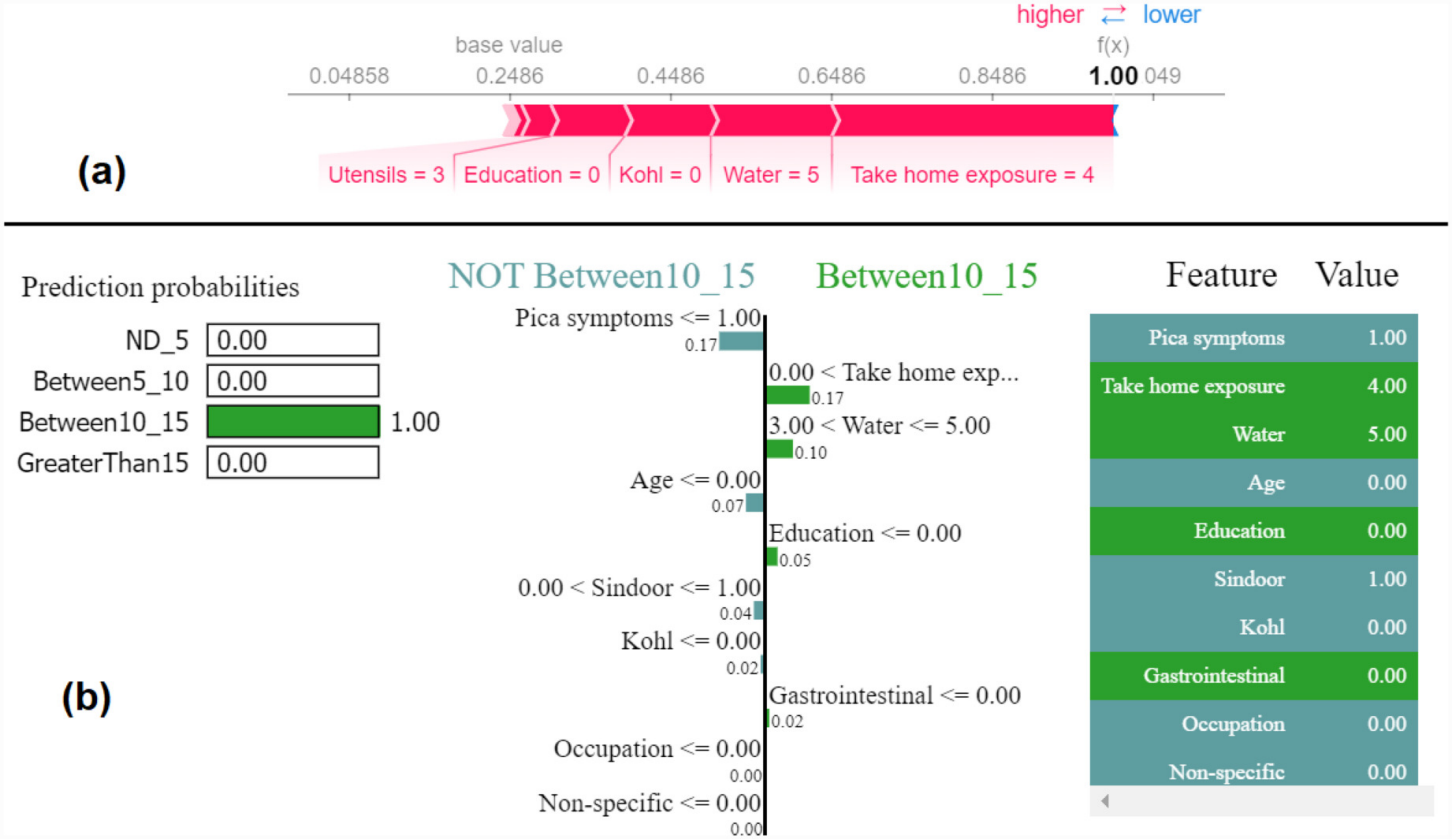
Example of the output class Between10_15: (**a**) Interpretation of model prediction results based on SHAP, (**b**) Explanation provided by the LIME model.

**Figure 10:**
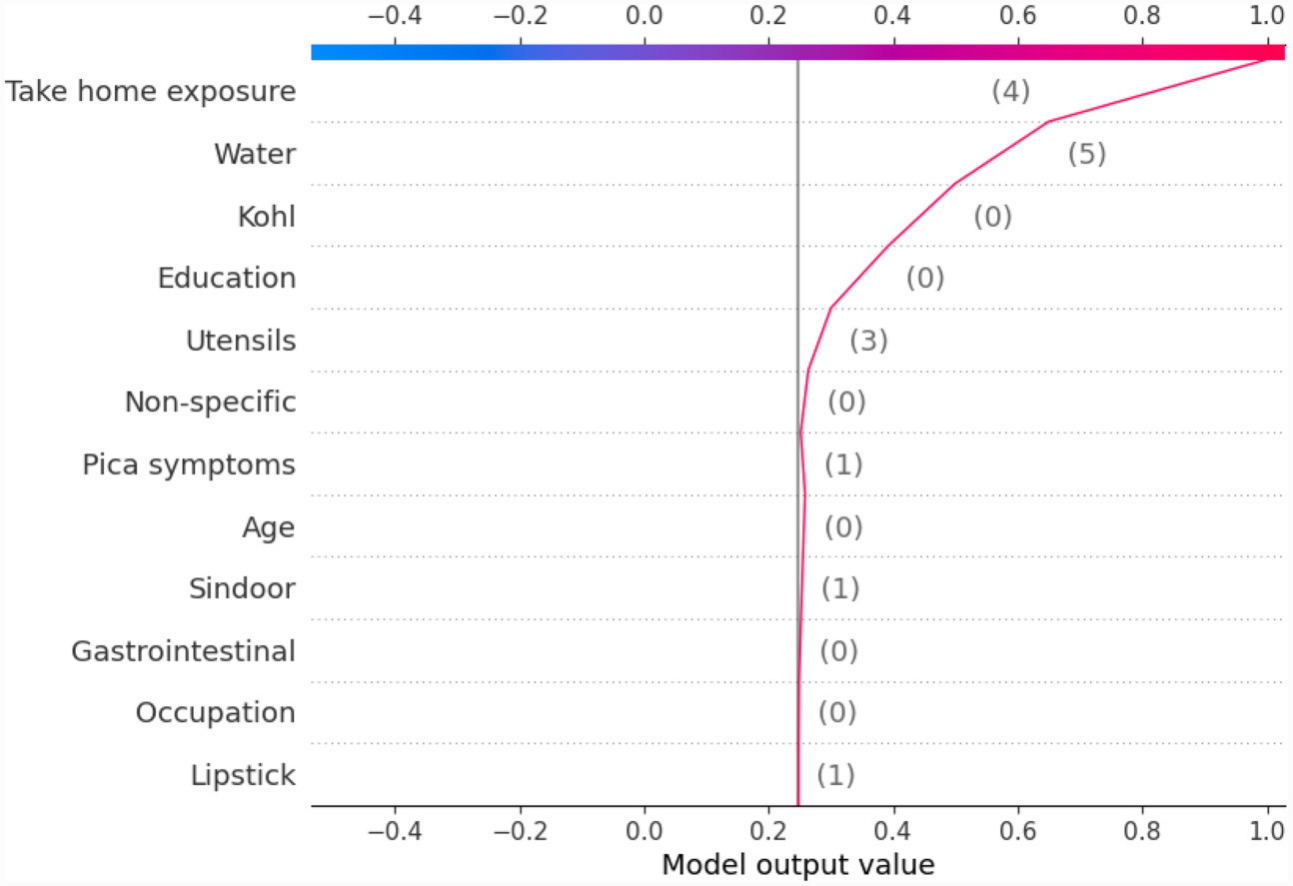
Example of SHAP decision plot for the output class Between10_15.

**Figure 11:**
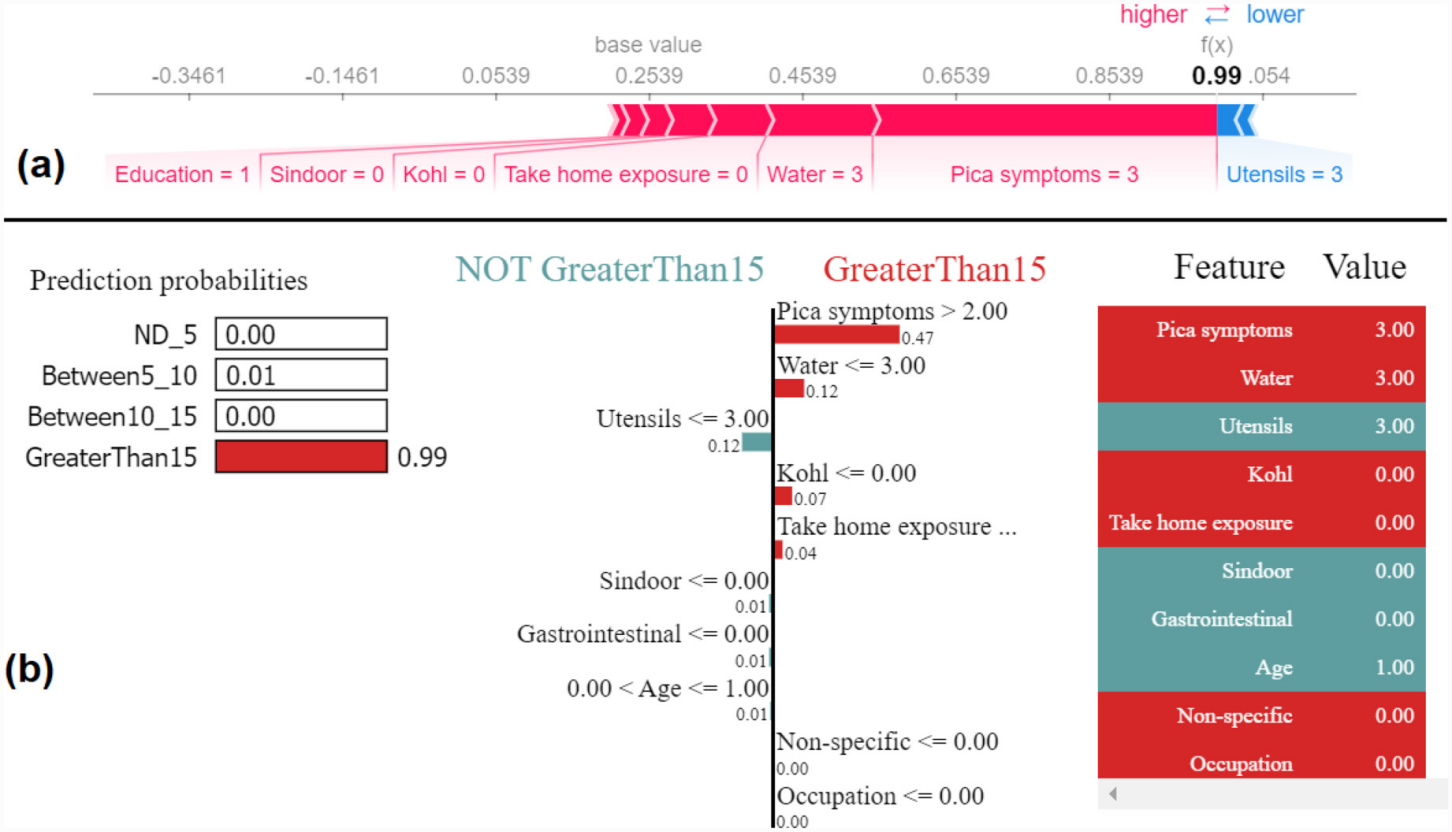
Example of the output class GreaterThan15: (**a**) Interpretation of model prediction results based on SHAP, (**b**) Explanation provided by the LIME model.

**Figure 12:**
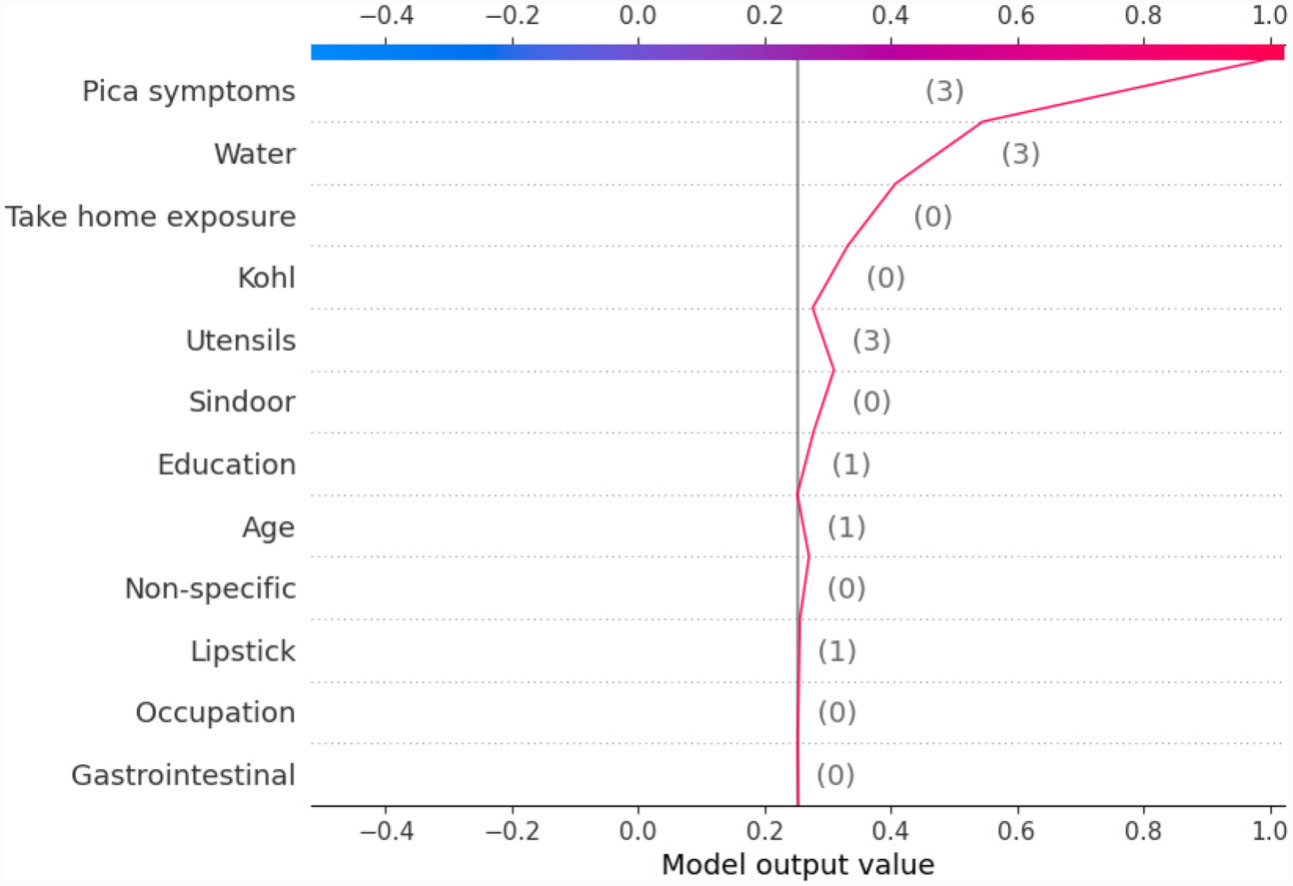
Example of SHAP decision plot for the output class “GreaterThan15.”

Similarly, we show SHAP and LIME plots for the other predicted class labels. Figures 7, 8 show the explanation for a given data observation for which the model predicted the class label as Between5_10. Figures 9, 10 show the explanation for a given data instance for which the model predicted the class label as Between10_15. Figures 11, 12 show the explanation for a given data instance for which the model predicted the class label as GreaterThan15.

**Figure 13:**
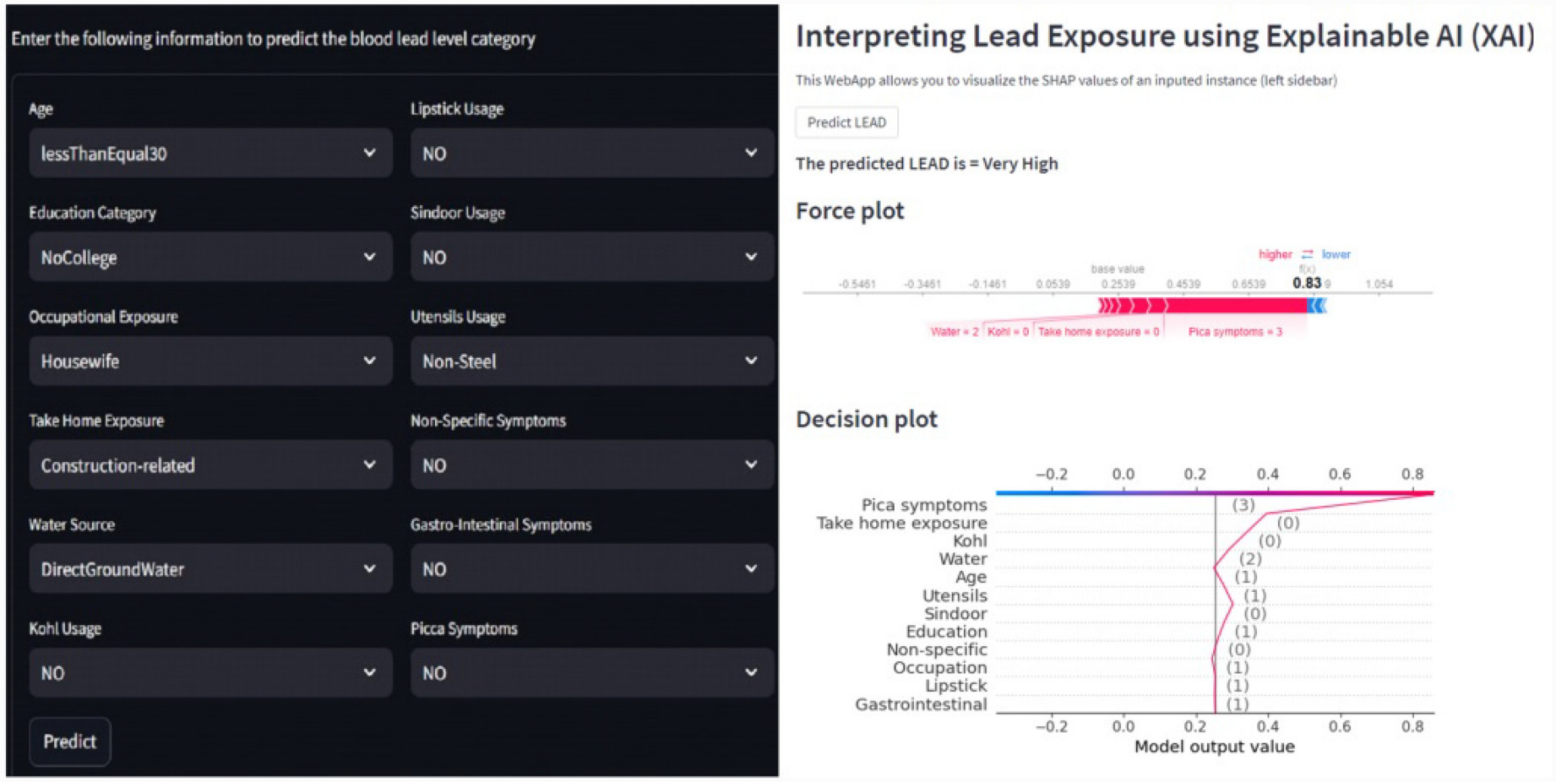
Web interface showing results for an input instance for which the predicted class is very high level (GreaterThan15) and the explanation of the outcome using the force and decision plots.

To compile all the results in one place, we also developed a Streamlit-based web interface. Streamlit is a Python library to create interactive web applications. Figure 13 shows the developed web interface showing results for an input instance for which the predicted class is very high level (GreaterThan15) and the explanation of the outcome using the force and decision plots. The interface can be useful in interpreting the results obtained without knowing the backend code. A non-technical user can enter the input values to the model through this interface and get the prediction result and explanations without manually running the backend code.

## 4 Discussion

In our initial work, with 12 features, the model was able to predict the lead toxicity levels. The built ML-based lead prediction model is a black box. The model can tell the lead toxicity level as low (ND_5), mild (Between5_10), high (Between10_15) and very high (GreaterThan15) for a given observation. However, if we want to reduce the impact of lead toxicity levels, we should be able to tell which features are highly contributing to the predicted lead levels in a particular subject. From the built model, we cannot make out to what extent each of these 12 features has contributed towards making a particular prediction. It is likely that not all the 12 features will equally contribute to the lead toxicity level. If we consider all the 12 features, it would be difficult to reduce the effect of all the 12 features and this may not be an effective approach. Therefore, if we know the most important features to focus on, it would be easier to reduce the risk of lead toxicity.

For the acceptance of AI in healthcare, these black-box models need to have transparency and explain the reasons for the predictions made. The XAI framework provides the medium for understanding the decisions made. Explaining the decisions made by the model will increase the trust and reliability of the built model. In our case, the end user of the lead screening tool is going to be healthcare professionals with usually a non-technical background. They should be able to understand the predictions made and interpret them. In this context, the work presented in this paper used the XAI framework to explain the model prediction and interpret the results. Using XAI, the prediction made by the model can be interpreted for decision-making, thus making the model equitable in real-world contexts.

In this paper, with the use of XAI, we are able to add an explanation to the prediction made. This is particularly important when we want to investigate modifiable risk factors that could be mitigated to reduce the effects of lead poisoning. Figure 3 indicated that the model picks pica symptoms as the top feature in determining the lead toxicity levels. This interpretation can be validated by analysing individual data points shown in Figure 4. For example, in Figure 4, for the class ND_5 if the pica symptoms value is low (green dots), the model predicts lower toxicity i.e., ND_5. On the other hand, for the class label GreaterThan15, if the pica symptoms value is high (blue dots), the model predicts very high lead toxicity i.e., GreaterThan15. Likewise, individual explanations can help in understanding why the model made specific predictions for a subject. The force plot gives insight and provides the magnitude of each feature contribution in making the prediction. For example, in Figure 11, for the given observation, the model has predicted GreaterThan15 toxicity level. The force plot gives the reason for this prediction. From Figure 11, it can be seen that pica symptoms, take-home exposure and water are the main reasons for this prediction. These factors can be worked upon to reduce the exposure in this particular subject.

## 5 Conclusion

Lead poisoning is very much preventable with effective screening and prevention programmes. Early identification of lead poisoning in the pregnant woman can help in reducing the harmful effects on the developing foetus. In our previous work, we were able to demonstrate the usability of the ML model in doing lead toxicity prediction based on sociodemographic features. Using XAI, the prediction made by the model can be interpreted for decision-making, thus making the model equitable in real-world contexts. In this work, with the use of the XAI framework, we were able to add the explainability layer to the built model. The XAI framework is applied to the RF black-box model to understand the decisions made by the model.

With the limited data of 200 samples, the work demonstrated the possibility of using easy-to-collect and non-invasive sociodemographic features for lead prediction modelling. The work is significant because we are applying ML techniques to explain and predict lead toxicity, which is determined mostly by using the traditional approach of lab testing. It demonstrates how lead toxicity prediction can be taken out of the lab and explored in a larger population with the limited resources available. Backed up by more data, in future, the work can be extended to provide greater speed and precision along with insights that can help healthcare providers plan and deliver care in the context of lead poisoning.

## Data Availability

The datasets presented in this article are not readily available because based on the ethical approval and GDPR requirements, the data is solely intended for the project purpose only. Requests to access the datasets should be directed to p.chaurasia@ulster.ac.uk.
